# Plant centromeric retrotransposons: a structural and cytogenetic perspective

**DOI:** 10.1186/1759-8753-2-4

**Published:** 2011-03-03

**Authors:** Pavel Neumann, Alice Navrátilová, Andrea Koblížková, Eduard Kejnovský, Eva Hřibová, Roman Hobza, Alex Widmer, Jaroslav Doležel, Jiří Macas

**Affiliations:** 1 Biology Centre of the Academy of Sciences of the Czech Republic, Institute of Plant Molecular Biology, Branišovská 31, České Budějovice CZ-37005, Czech Republic; 2 Institute of Biophysics of the Academy of Sciences of the Czech Republic, Královopolská 135, Brno CZ-61265, Czech Republic; 3 Laboratory of Molecular Cytogenetics and Cytometry, Institute of Experimental Botany of the Academy of Sciences of the Czech Republic, Sokolovská 6, Olomouc CZ-77200, Czech Republic; 4 ETH Zurich, Institute of Integrative Biology, Universitätstrasse 16, CH-8092 Zürich, Switzerland

## Abstract

**Background:**

The centromeric and pericentromeric regions of plant chromosomes are colonized by Ty3/gypsy retrotransposons, which, on the basis of their reverse transcriptase sequences, form the chromovirus CRM clade. Despite their potential importance for centromere evolution and function, they have remained poorly characterized. In this work, we aimed to carry out a comprehensive survey of CRM clade elements with an emphasis on their diversity, structure, chromosomal distribution and transcriptional activity.

**Results:**

We have surveyed a set of 190 CRM elements belonging to 81 different retrotransposon families, derived from 33 host species and falling into 12 plant families. The sequences at the C-terminus of their integrases were unexpectedly heterogeneous, despite the understanding that they are responsible for targeting to the centromere. This variation allowed the division of the CRM clade into the three groups A, B and C, and the members of each differed considerably with respect to their chromosomal distribution. The differences in chromosomal distribution coincided with variation in the integrase C-terminus sequences possessing a putative targeting domain (PTD). A majority of the group A elements possess the CR motif and are concentrated in the centromeric region, while members of group C have the type II chromodomain and are dispersed throughout the genome. Although representatives of the group B lack a PTD of any type, they appeared to be localized preferentially in the centromeres of tested species. All tested elements were found to be transcriptionally active.

**Conclusions:**

Comprehensive analysis of the CRM clade elements showed that genuinely centromeric retrotransposons represent only a fraction of the CRM clade (group A). These centromeric retrotransposons represent an active component of centromeres of a wide range of angiosperm species, implying that they play an important role in plant centromere evolution. In addition, their transcriptional activity is consistent with the notion that the transcription of centromeric retrotransposons has a role in normal centromere function.

## Background

Long terminal repeat (LTR) retrotransposons represent a common class of mobile genetic elements in eukaryotic genomes [1-7]. Because of their replicative mode of transposition based on an RNA intermediate, they compose the majority of the DNA of many eukaryotic genomes. They are particularly abundant in plant genomes and are intimately involved in the evolution of genome structure and size [8,9]. Plant retrotransposon families differ considerably from one another, not only with respect to their sequence and structure but also with regard to their chromosomal distribution. Thus, while some plant retrotransposon families are essentially randomly dispersed, others are concentrated in distinct chromosomal regions [10,11]. Among the latter category are the centromeric retrotransposons, which accumulate preferentially in the centromeric region. (Note that the term "centromeric" is used hereinafter to refer to both the centromeric and pericentromeric regions, as these are difficult to distinguish from one another.) They usually accompany arrays of satellite DNA, which are the dominant centromeric sequences in most species [12]. However, centromeres of some species, such as wheat [13], are dominated by centromeric retrotransposons.

A number of centromeric retrotransposons have been fully characterized in grass species: specifically, RIRE7 and CRR in rice (*Oryza sativa*) [14-17], CRM in maize (*Zea mays*) [18,19], CRW in wild einkorn wheat (*Triticum boeoticum*) [13], CRS in sugar cane (*Saccharum officinarum*) [20], Bilby in cereal rye (*Secale cereale*) [21] and Cereba in barley (*Hordeum vulgare*) [22,23]. In sorghum (*Sorghum bicolor*), pHind22 and pSau3A9 have been partially characterized [24]. Equivalent elements extracted from dicotyledonous species include Beetle1 (sugar beet, *Beta vulgaris*) and Beetle2 (wild beet, *Beta procumbens*) [25,26] as well as CRA (*Arabidopsis thaliana*, hereinafter referred to as *At*) [27,28]. Their phylogeny, based on their reverse transcriptase (RT) sequences, reveals that they are chromoviruses (*Chromoviridae*), a lineage of Ty3/gypsy retrotransposons possessing an integrase chromodomain [27,29,30]. Further classification of chromoviruses has shown that these centromeric retrotransposons form a phylogenetically distinct clade designated CRM [27,29,30]. Although the chromoviruses are widespread within eukaryotic genomes, CRM elements are specific to plants, both angiosperms and gymnosperms [27]. Few of these elements have been described in any detail, and little is known of their chromosomal distribution. Thus it remains unclear both whether all CRM elements are in reality centromeric retrotransposons and how widespread the genuine centromeric retrotransposons are in plant genomes.

The most distinctive structural feature of a centromeric retrotransposon is the presence of an integrase chromodomain, which is widely assumed to ensure correct targeting to the centromeric region [30]. Although chromodomains are present at the integrase C-terminus in all chromoviruses, their sequence is highly polymorphic [27,29-31]. On the basis of their similarity to cellular chromodomains (for example, those present in HP1 or Swi6 proteins), chromovirus chromodomains have been classified into types I and II and a CR motif [31]. Types I and II chromodomains have sequence and structural similarity both to cellular chromodomains and to each other. However, while the type I chromodomains contain all three aromatic residues known to recognize methylated lysine on histone H3 (H3K9), type II chromodomains lack the first and usually also the last of these residues. Unlike all other plant chromoviruses which include a type II chromodomain, centromeric retrotransposons possess a CR motif, which is key for the recognition of centromeric chromatin [31]. Although the CR motif is found at the position corresponding to a chromodomain, Gao *et al*. [31] showed that it has neither sequence nor structural similarity to types I and II chromodomains, suggesting that it is not a genuine chromodomain. For this reason, all sequences found at the position of a chromodomain are collectively referred to hereinafter as putative targeting domains (PTDs). Although the CR motif's interacting partner has yet to be identified, it has been established that, unlike the type I chromodomains, it involves neither a dimethylated nor a trimethylated form of histone H3 lysine 9 (H3K9me2, H3K9me3) [31].

Circumstantial evidence suggests that centromeric retrotransposons have been influential in the evolution of centromeres, as well as in their structure and function. Their transpositional activity contributes to high evolutionary dynamics of centromeres by generating new insertions, which may be further subjected to illegitimate and unequal homologous recombination [32,33]. Transcription driven by centromeric retrotransposon promoters has been proposed to underlie the substitution of histone H3 by CenH3 (centromere-specific variant of histone H3 which is essential for the establishment and maintenance of centromere function and kinetochore assembly) [12]. As the RNA component of maize centromeric chromatin includes CRM retrotransposon transcripts, it has been suggested that centromeric retrotransposons are also important determinants of the structure of centromeric chromatin [34]. Because transcripts of CRR elements are processed by the RNA interference (RNAi) machinery of rice, Neumann *et al. *[35] proposed that these elements play a role in RNAi-mediated formation and maintenance of centromeric chromatin. However, as yet there have been no systematic attempts to investigate the function of centromeric retrotransposons in centromere activity, largely because of a lack of sufficient representatives to build a generalized picture that is valid across a spectrum of plant species. Thus, here we set out to produce a comprehensive survey of plant CRM retrotransposons. We have analyzed their nucleotide and protein sequences, with a goal of illuminating their structure, diversity, type of PTD, chromosomal distribution and transcriptional activity.

## Results

### Identification of putative centromeric retrotransposon sequences

**Figure 1:**
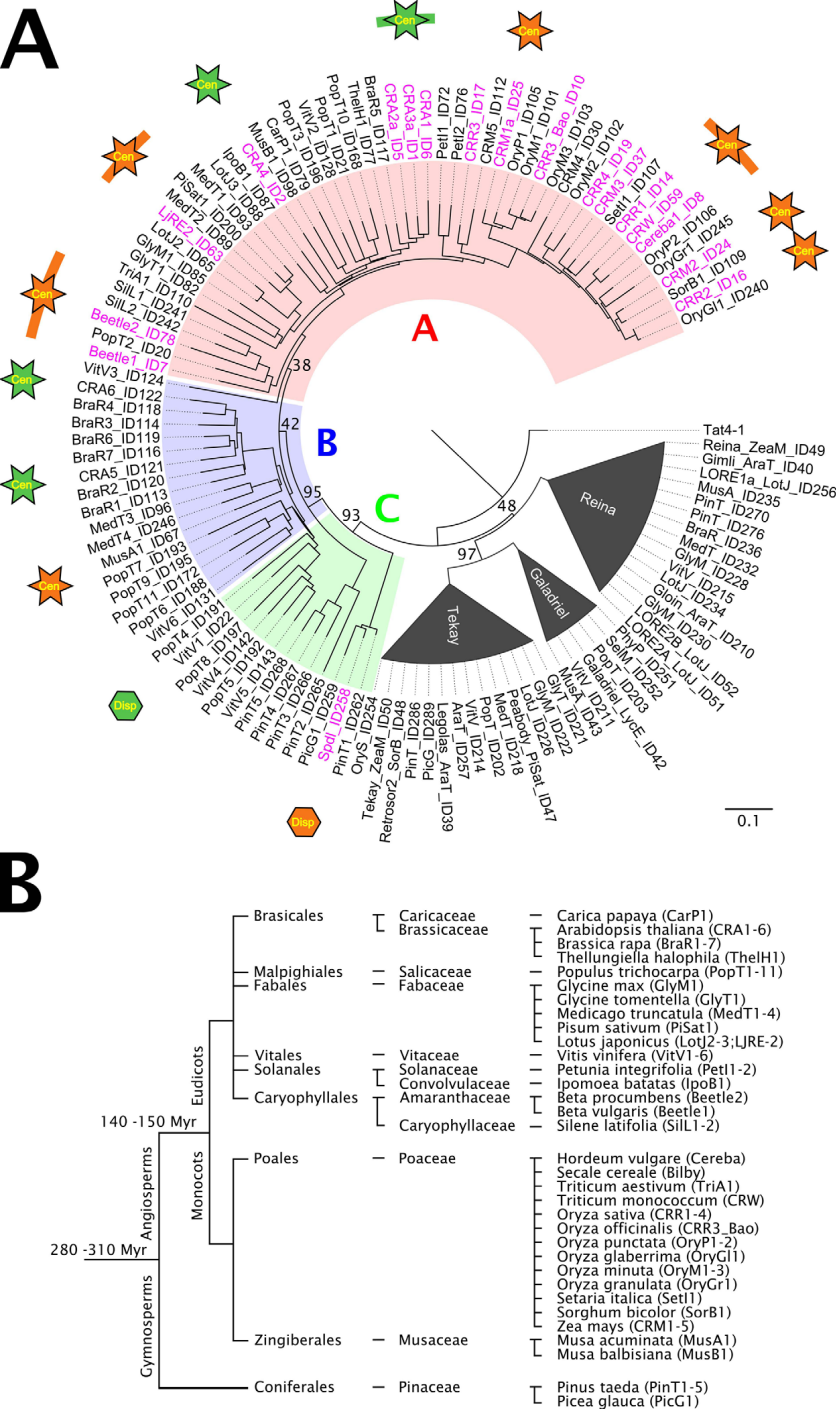
**Diversity of CRM families and their species of origin**. **(A) **Neighbor-joining tree inferred from a comparison of reverse transcriptase (RT) domain sequences. The non-chromovirus element Tat4-1 was used as an outgroup, while members of the Tekay, Reina and Galadriel clades were included as representatives of other plant chromoviruses. Alignment of the RT domains is provided in Additional file 3: Alignment of RT domains. On the basis of differences at the C-terminus of integrase, the CRM families were divided into groups A, B and C (Figure 3). Previously described CRM members are shown in purple (see also Additional file 1: Origin and structural features of sequences used in this work). Families with confirmed centromeric localization are marked with orange stars (fluorescence *in situ *hybridization results) or green stars (*in silico *localization). Families having a dispersed chromosomal distribution are labeled with orange or green hexagons. Bootstrap values are shown only for the major nodes. Elements belonging to the Tekay, Reina and Galadriel clades are listed in Additional file 1: Origin and structural features of sequences used in this work. It should be noted that because of the limitations of the neighbor-joining method and the lack of representatives from a wider range of evolutionarily distant species, the tree topology may not fully reflect real phylogenetic relationships between different groups of CRM elements. **(B) **Taxonomy classification of the species containing the CRM elements. Dates of divergence between major groups of plants are from the work by Chaw *et al*. [105]. The names of CRM families present in the species are shown in brackets.

The *in silico *search for CRM elements detected 145 novel elements, which fell into 63 families on the basis of species of origin and sequence similarity. An additional three families were identified from the sequence contigs assembled from the 454 derived sequences of pea and white campion. In addition to the sequences described in the literature, we gathered 190 elements representing 81 different retrotransposon families and distributed across 33 plant species belonging to 12 plant families (Figure 1; see also Additional file 1: Origin and structural features of sequences used in this work, and Additional file 2: CRM sequences used in this study). The phylogenetic analysis of representatives of each of the families, based on their RT domain sequence, clustered all the *de novo *sequences with previously identified CRM members (Figure 1A; see also Additional file 3: Alignment of RT domains). The same result was obtained by extending the analysis to a comparison of integrase and whole polyprotein sequences, confirming the appropriateness of the RT domain sequence (data not shown).

### Elements belonging to the CRM clade are variable at their integrase C-terminus

**Figure 2:**
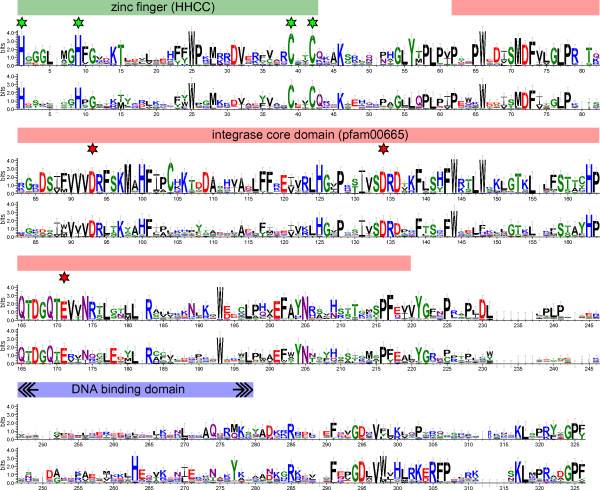
**Graphical representation of the conserved portion of the integrase protein sequence**. Integrase sequences extracted from CRM, Tekay, Reina and Galadriel chromoviruses aligned using the Muscle program are shown as sequence logo plots [96]. CRM clade members are shown in the upper part of the figure, and those from the other clades are shown in the lower part. Despite the overall high level of sequence similarity, several amino acid residues are conserved only within the CRM clade. The HHCC and DD35E motifs are indicated by green and red stars, respectively.

**Figure 3:**
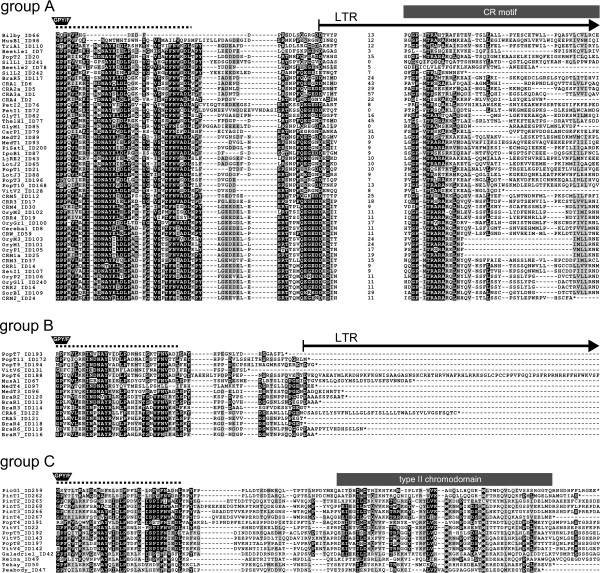
**Alignments of sequences at the C-terminus integrase**. Group A elements possess a CR motif with several strongly conserved amino acid residues near the N-terminus. These residues are not present in Beetle1, Beetle2 or SilL1, but are well conserved in the grass species elements (see bottom part of the alignment). The numbers between two aligned blocks specify the number of amino acid residues not shown. No putative targeting domain (PTD) is encoded by group B elements. The type II chromodomain of group C elements shares sequence similarity with Tekay, Galadriel and Reina clade members. The dotted line above each alignment marks a region conserved among all plant chromoviruses. The arrow shows the portion of the integrase lying within the 3' long terminal repeat(LTR). Asterisks indicate stop codons at the end of each open reading frame. A highly conserved GPY/F motif [36,37] is indicated by a black trapezoid above the beginning of each alignment.

The integrase protein is probably critical for the correct targeting of the centromeric retrotransposons to the centromere region. Most of the CRM integrases possessed a zinc finger with an HHCC motif at its N-terminus and a core domain containing the D,D(35)E motif around the active site (Figure 2). Between the core domain and the C-terminus, which presumably includes the DNA binding region and PTD, sequence divergence prevented full alignment. While the putative DNA binding region contained several strongly conserved amino acid residues, the PTDs and their flanking sequences were variable. Surprisingly, this also applied to the CR motif, which was relatively well conserved in previously described elements, except for Beetle1 and Beetle2 [26,31]. Of 81 CRM clade families, only 50 showed similarity to the CR motif. The integrases of the remaining families either possessed a type II chromodomain in place of the CR motif or lacked a PTD of any type. On the basis of the presence or absence and type of PTD, the elements were divided into three groups (Figures 1A and 3).

Group A members possessed the CR motif, although in a few cases the motif was significantly altered (Figure 3). Apart from Beetle1 and Beetle2, the most mutated CR motifs occurred in SilL1 and SilL2. Comparison of these elements with 454 sequencing-generated reads containing partial sequences of SilL1 and SilL2 showed high protein similarity in this region, suggesting that the altered sequences of the CR motif in these subfamilies were most likely due not to mutations in the two analyzed sequences but rather to a real divergence of these families from other elements belonging to group A.

Integrase sequences of the group B elements lacked any PTD, and they terminated shortly beyond the conserved glycine-proline-tyrosine/phynelyalanine (GPY/F) motif [36,37] (Figure 3). To confirm that the absence of PTD was not an *in silico *translation error, evidence for the presence of the CR motif or the type II chromodomain typical for all other plant chromoviruses was sought within predicted polypeptides translated in all possible reading frames. Although these searches involved using the BLASTP, RPS-BLAST and MAST programs (National Center for Biotechnology Information (NCBI), http://www.ncbi.nlm.nih.gov/) to maximize sensitivity, the results were consistently negative. Together with their intact coding region and the similarity shown by the polyprotein termini, the evidence therefore strongly suggested that these groups of chromoviruses encode neither the type II chromodomain nor the CR motif.

Elements encoding the type II chromodomain were defined as group C (Figure 3). Similarly to other plant chromovirus clades (Tekay, Reina, Galadriel), the chromodomain of group C elements lacked the conserved aromatic cage residues known to interact with methylated H3K9. It should be noted that among members of this group were found all gymnosperm sequences, some of which were highly similar to the partial chromodomain-lacking sequence of the Spdl element [GenBank:AF229251] [38], present in white spruce (*Picea glauca*) and classified as a CRM member by Gorinsek *et al. *[27].

### Structural features of the CRM elements

**Figure 4:**
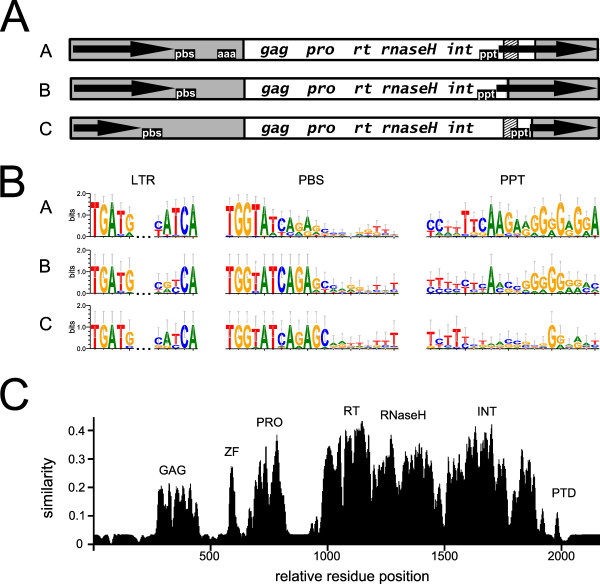
**Structural analysis of CRM elements**. **(A) **Polyprotein coding (white boxes), noncoding (gray boxes), putative targeting domain (PTD) (hatched boxes) and long terminal repeats (LTRs) (arrowed). Pbs, primer binding site; ppt, polypurine tract; aaa, A-rich stretch. The group A member coding region extends into the 3' LTR, which encodes the CR motif. Group B elements lack any PTD. Group C possesses a type II chromodomain-coding domain which terminates close to the 5' end of the 3' LTR. The graph is not drawn in proportion to segment lengths in base pairs. **(B) **Most elements share TGATG and T/CATCA inverted repeats at, respectively, the 5' and 3' end of the LTR. The primer binding site complementary to the 3' end of tRNA^Met ^differs in sequence between various families. Group A elements contain highly conserved polypurine tract sequences. **(C) **A protein similarity plot shows that the CRM polyproteins are highly conserved, varying mainly within their C-terminal PTD regions. Individual polyprotein domains: GAG, capsid domain, similar to pfam37032; ZF, nucleocapsid GAG protein zinc finger; PRO, protease; RT, reverse transcriptase RNase H; IN, integrase; PTD, putative targeting domain.

The range in size of the complete CRM elements was approximately 5.1 to 10.2 kbp. They were flanked by two LTRs ranging from 299 to 1,225 bp. The LTR termini featured the highly conserved inverted repeat motif 5'-TGATG/CATCA-3'. Upon insertion, CRM elements generated a 5-bp target site duplication, the sequence of which varied substantially from insertion to insertion. Thus these elements do not appear to target specific sequences in the genome. The age of the insertions ranged from 0 to 6.7 million years ago (see Additional file 1: Origin and structural features of sequences used in this work), demonstrating the recentness of insertion activity of CRM elements. The 5' LTR was followed by a primer binding site, while the 3' LTR was preceded by a polypurine tract (Figures 4A and 4B). Although the primer binding site of all CRM subfamilies was complementary to 12 to 18 nucleotides at the 3' end of tRNA^Met^, its sequences were only partially conserved, corresponding to various types of tRNA^Met ^(Figure 4B). The polypurine tract ranged in length from 4 to 13 bp, and its sequence in group A elements was highly similar even between distantly related species (Figure 4B; see also Additional file 1: Origin and structural features of sequences used in this work). The A-rich stretch within the 5' UTR, common to many rice CRRs [17], was present in a number of group A elements (including those present in dicotyledonous species), which suggests its likely importance as a structural feature. However, it was absent in most members of groups B and C. The polyprotein region extended into the 3' LTR in all group A elements, but only in a few elements of groups B and C (Figure 4A). Although the coding sequence was interrupted by nonsense codons and/or frame shifts in many elements, it seemed to be organized as a single open reading frame in the intact autonomous elements. The putative polyprotein sequences contained all the domains necessary for replication and integration (gag, protease, RT, RNase H and integrase) (Figure 4A), showing a pronounced level of similarity between elements (Figure 4C). A relatively high level of similarity was also found between nucleotide sequences of the elements (see Additional file 4: Dot plot comparison of full-length CRM elements).

### Not all retrotransposon families within the CRM clade are accumulated in centromeres

**Figure 5:**
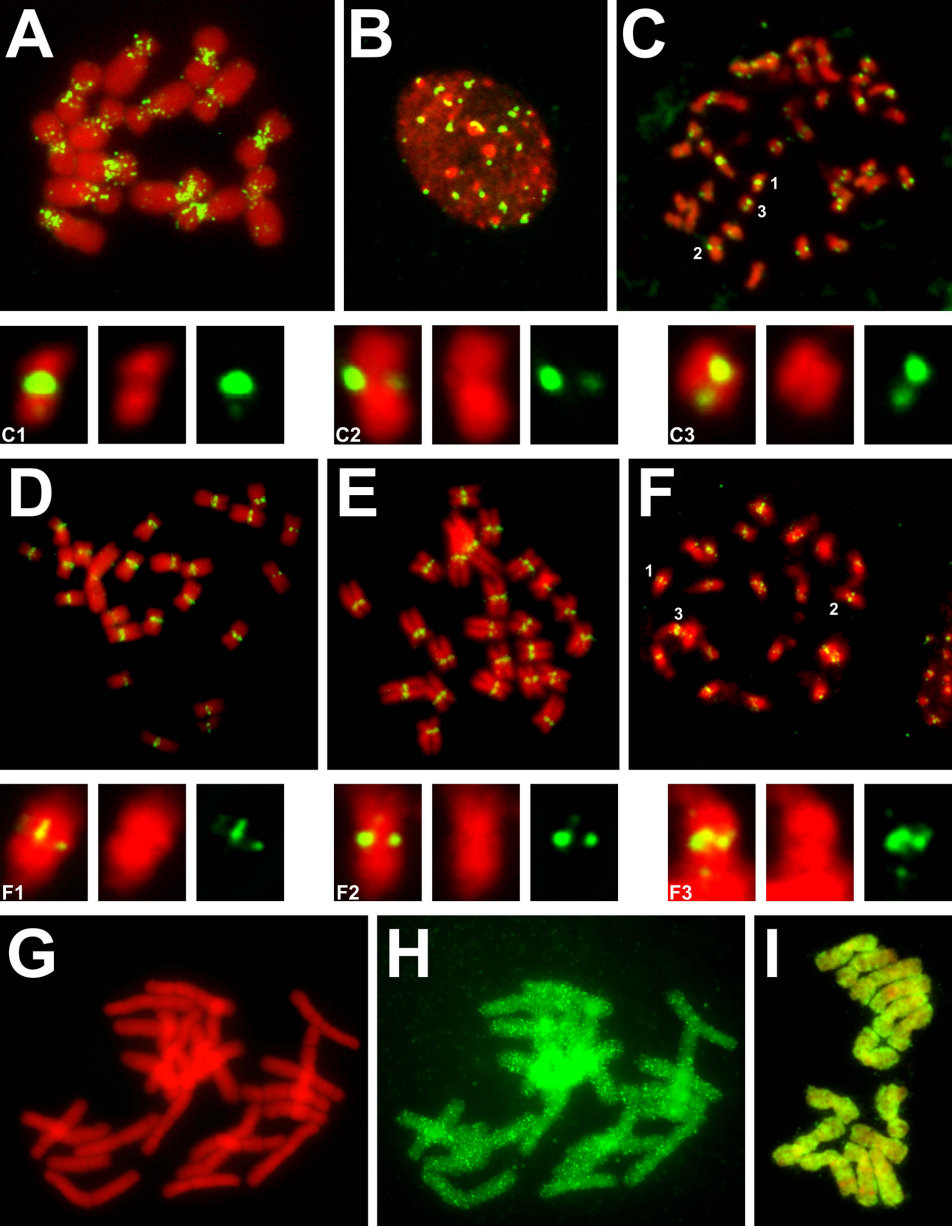
**Fluorescence *in situ *hybridization (FISH)-based visualization of the intrachromosomal distribution of chromoviruses**. **(A) **Pea chromosomes hybridized with PiSat1 (group A). **(B and C) **Black cottonwood interphase nucleus and metaphase chromosomes hybridized with PopT2 (group A). Note that most of the signal is associated with chromocenters (bright 4',6-diamidino-2-phenylindole (DAPI)-stained spots in the interphase nucleus). Three metaphase chromosomes were enlarged to allow a clearer localization of PopT2 to the centromeric region (C1-C3). **(D and E) **White campion chromosomes hybridized with SilL1 and SilL2 (group A). **(F) **Banana chromosomes hybridized with MusA1 (group B). Since all of the banana chromosomes are metacentric or submetacentric, signals located around the center of the chromosome are taken to reflect loci near or within the centromere. Three of the chromosomes with identifiable centromeres were enlarged (F1-F3). **(G and H) **Norway spruce chromosomes counterstained with DAPI and hybridized with a Spdl-like sequence (group C). **(I) **Pea chromosomes hybridized with Peabody (Tekay clade). Positive hybridization signals are shown in green, and DAPI-counterstained DNA appears in red.

Although the elements described above formed a well-defined phylogenetic clade, it remained to be established whether they were all preferentially localized in centromeric regions. The chromosomal distribution of selected families was investigated both experimentally by fluorescence *in situ *hybridization (FISH) and computationally in those species in which the whole genome sequence was available. A centromeric FISH signal was observed for all of the group A sequences tested (including PiSat1 in pea, SilL1 and SilL2 in white campion and PopT2 in black cottonwood) (Figure 5). A weak MedT1/2 centromeric signal was observed in barrel medic (data not shown). No detectable VitV2 FISH signal was obtained in grape, a result ascribable to a copy number of only approximately 50 per haploid genome, according to both a dot blot hybridization experiment and an *in silico *search of the whole grape genome sequence. The distribution of rare VitV2 copies in the whole genome sequence was essentially random (data not shown), but it must be borne in mind that published chromosome sequences are still incomplete and the position of the centromeres is as yet ill-defined [39].

Surprisingly, we demonstrated by FISH with MusA1 in banana that even elements lacking PTD (group B members) can be preferentially accumulated in centromeres (Figure 5F). *At *elements CRA5 and CRA6, other representatives of group B, were also found in centromeres, but it should be mentioned that they have only a few copies in the genome (data not shown).

On the other hand, two tested representatives of group C showed dispersed distribution along chromosomes. The distribution of VitV1 elements in the current genome assembly appeared random (data not shown), a result which could not be validated cytogenetically, as the copy number of this element is too low (100 to 500 copies per haploid genome). When FISH was attempted in Norway spruce using a probe which shared 96% and 94% identity with white spruce Spdl and PicG1 sequences, respectively, the hybridization signal was dispersed along the whole length of all chromosomes (Figures 5G and 5H), unlike the pattern generated after probing with the centromeric satellite 2F [40] used to label centromeric regions (data not shown).

The same *in silico *strategy was extended to investigate the intrachromosomal distribution of elements belonging to other chromovirus clades represented in the complete *At *and rice genome sequences. While non-CRM chromoviruses are concentrated in the centromeric region of *At*, those in rice appear to be dispersed throughout the genome (data not shown) [31]. The distinct chromosomal distribution of rice and *At *type II chromodomain-containing chromoviruses, in combination with our own unpublished findings from preliminary experiments performed in other species as well as other data in the literature, suggest that the distribution of the elements may correlate with genome size. Therefore, we also carried out FISH based on a fragment of the pea retrotransposon Peabody, which is the most abundant chromovirus family (Tekay clade) in this species, with a copy number of about 10,000 per haploid genome (4,300 Mbp/1C) [41-43]. The hybridization signal covered every chromosome almost uniformly, although it was absent from secondary constrictions and major heterochromatic blocks (Figure 5I).

### Elements possessing the CR motif are common in the angiosperms

As the search for novel CRM retrotransposons was aimed only at full-length sequences predicted by the LTR_Finder program (http://tlife.fudan.edu.cn/ltr_finder/), it excluded partial elements. An attempt was made to widen the search by trawling GenBank for species not identified by the initial search, querying with a set of all polyprotein domains extracted from the chromovirus elements shown in Figure 1A. This generated a set of >100 sequences showing >70% identity to CRM representatives (data not shown) and originating from both angiosperm and gymnosperm species. The angiosperm sequences were related to representatives of all three groups. However, all of the gymnosperm sequences were of the group C type. While the C-terminal portion of the integrase gene bearing the CR motif was present in a broad range of angiosperm species, it was not detected in any nonangiosperms. Thus, group A elements are either angiosperm-specific or have not been sequenced yet in gymnosperms.

### The CRM elements are transcribed

A growing body of evidence suggests that noncoding transcripts derived from centromeric repetitive DNA, as well as small RNA produced via their RNAi-mediated degradation, are important for the proper function of the centromere [34,44,45]. When reverse-transcriptase polymerase chain reaction (RT-PCR) was performed to assay the transcriptional activity of a number of centromeric retrotransposon families, amplicons of the appropriate length were recovered in every case. Thus, transcriptional activity appears to be a general feature of centromeric retrotransposons (Additional file 5: Transcription of centromeric retrotransposons). A database search for small RNA sequences in *At *[46], barrel medic [47] and black cottonwood [48], as well as in pea (using an in-house database) identified small RNA sequences matching CRM clade elements in each of these species. With the exception of black cottonwood, for which very little sequence data were available (about 27,000 sequences, 14 of which were identical to PopT retrotransposons), >100 distinct small RNA sequences per species were identified. The abundance of particular small RNA was estimated on the basis of the frequency with which they occurred in each library, and this proved to be very low: only one or a few per tens of thousands to several million (data not shown). The global frequency of small RNA was low as well, especially in pea and barrel medic (respectively, 8 and 13 transcripts per quarter million, TPQ). The highest global frequency was found in *At *siliques (449 TPQ; see Additional file 5: Transcription of centromeric retrotransposons). The size range of these small RNA was 18 to 27 nt, but most were 24 nt (Additional file 5: Transcription of centromeric retrotransposons), and they originated from throughout the whole element sequence. In *At*, centromeric retrotransposon small RNA were represented in four different tissues, suggesting that they are constitutively transcribed and that RNAi is involved in their processing. A similar number of small RNA sequences were also present in *At *mutant lines in which the activity of various RNAi genes was disrupted (Additional file 5: Transcription of centromeric retrotransposons).

## Discussion

### The classification of CRM retrotransposons

Since centromeric retrotransposons have been classified as belonging to the chromovirus CRM clade [27,29,30], it was expected that the elements identified here would be largely concentrated in the centromeric region. Although the members of the CRM clade are taken as being the most highly conserved of the plant chromoviruses [27], the present data showed that they do vary sufficiently to allow for their subdivision into three groups distinguished with respect to the structure of their integrase C-termini (Figure 3). The CR motif, shown by Gao *et al. *[31] to be particularly well conserved, was indeed present in most of the group A members, but was lacking in those from groups B and C. Group C elements possessed a type II chromodomain in the place of the CR motif, while those in group B appeared to lack any kind of PTD. Although real phylogenetic relationships between elements from different groups remain to be resolved, the presence of the type II chromodomain in group C elements probably reflects an evolutionary divergence between the CRM and the other plant chromovirus clades from a common ancestor possessing this type of PTD. On the other hand, group B elements probably derived from those belonging to groups A and/or C either by deletion of PTD-coding region or by successive accumulation of mutations.

### CRM clade members are not confined to the centromeric region of plant chromosomes

Although they appeared to be phylogenetically closely related to one another, CRM clade members were not universally localized to the centromeric region of the chromosome. The Spdl-like sequence (group C) in particular was dispersed over the whole length of the Norway spruce genome. A second CRM clade member with this type of genomic distribution was VitV1 (group C), although the evidence supporting its dispersed nature has relied entirely on an assembly of the grape genome known to be as yet incomplete [39]. Such an intrachromosomal distribution is consistent with that of other chromovirus non-CRM families containing the same type of PTD, such as Peabody in pea (Figure 5I) and a Peabody-like sequence in white campion [49]. On the other hand, group B elements, although they lack a PTD, tended to be concentrated in the centromeric region. Whether this has come about because of a PTD-independent targeting mechanism or whether group B elements accumulate in the centromeric region via some other process remains unclear.

### What makes centromeric retrotransposons centromeric?

The general assumption is that the PTD is responsible for the targeting of centromeric retrotransposons to the centromeric region. Experimental evidence for this targeting by the CRM PTD has been generated in *At *[31]. Chromatin immunoprecipitation-based experiments have demonstrated that centromeric retrotransposons are associated with histones CenH3 and H3K9me2 [13,17,19,35,50] and are depleted in the euchromatic fraction marked with H3K4me2 [35]. While the interaction with CenH3 has yet to be tested, it has been demonstrated that the CRM PTD does not interact with H3K9me2 [31]. Provided that the integrase C-terminus ensures centromere-specific integration, it is reasonable to assume that the CR motif is a key component of the targeting process, since this is the sole relatively well-conserved portion of an otherwise rather variable sequence (Figure 3). This line of argument is challenged, however, by the centromeric localization of plant Ty3/gypsy retrotransposons lacking the CR motif. These include CRM group B members in addition to, in *At *at least, representatives of three major Ty3/gypsy retrotransposon lineages, two of which (Tat and Athila) lack any sort of PTD [51,52]. Some chromoviruses possessing the type II chromodomain, especially those belonging to the Tekay clade, are concentrated in the centromeric regions of *At *[31] and banana [53]. In contrast, chromoviruses possessing type II chromodomains are dispersed along the chromosome arms in rice [31]. Peabody (Tekay clade) and PIGY (Athila lineage) elements are both highly dispersed in pea [54] (Figure 5I). Relatives of these two families are also dispersed in white campion [49]. Thus, while centromeric localization is the norm for elements possessing the CR motif, that of elements from lineages or clades lacking the CR motif is less predictable, although there is a tendency for their dispersion to be favored in large genomes. Heterochromatin in small genomes, as defined by the presence of methylated H3K9, is localized principally in the centromeric region, while in larger genomes, heterochromatic sites occur along the length of the chromosomes [55]. As a result, the apparently inconsistent intrachromosomal distribution of elements with particular types of PTD may simply reflect the contrasting distribution of heterochromatin. A consequence of this model is that elements possessing the CR motif must be able to recognize a centromeric chromatin-specific mark, while those with a type II chromodomain recognize a mark specific to heterochromatin more generally. No experimental evidence is available yet to either support or refute this notion, nor has any mechanism been suggested which can explain the colonization of the centromeric regions by elements that lack a PTD. However, a previous study of *At *showed that the accumulation of retrotransposons in centromeres may be the result of not only targeting but also purifying selection from centromere distal regions [51]. For the time being, therefore, we suggest that the term "centromeric retrotransposons" be reserved for group A elements, because only these are likely to actively target the centromeric region.

### How widespread are the centromeric retrotransposons?

The present data show that CRM retrotransposons are widespread among seed plants. However, representatives of groups A and B were present in the angiosperms (both mono- and dicotyledonous species), but not in the gymnosperms and evolutionarily older species, such as the moss *Physcomitrella patens*, the genome of which has recently been sequenced [56,57]. All CRM elements with confirmed centromeric localization belong to one or the other of these two groups. Thus, genuinely centromeric retrotransposons are either angiosperm-specific or are yet to be discovered in the other groups of plants. The gymnosperm CRM elements that we have identified belong to group C and are noncentromeric. Note that the *Pinus pinaster *pPpgy1 sequence was wrongly cited by Gorinsek *et al. *[27] as being centromeric ([58] and J.S.P. Heslop-Harrison, personal communication), and we believe that it is more likely to be a member of another chromovirus clade. Some insertions have proven to be very recent. The maintenance of transpositional activity suggests that CRM clade members are probably not all mere relics of earlier activity. The degree of amplification in the host genome differs from retrotransposon family to retrotransposon family. For instance, the copy number of *At *CRA is small, while that of the Norway spruce Spdl-like sequence reaches 50,000 to 100,000. A combination of published data for rice and maize [17,28], along with the present data relating to banana, white campion, pea, grape and black cottonwood, indicates that the copy number of group A and B members, which is in the range of hundreds to a few thousand, is lower than that achieved by at least some group C families.

### The role of centromeric retrotransposons in centromere function

Whether centromeric retrotransposons play any role in centromere function is of fundamental interest. One possibility is that they are merely parasitic and target the centromeric region to escape negative selection against insertions in distal regions of the chromosome [31]. The opposing hypothesis holds that they play a positive role in centromere function [59], in which case their targeting is also beneficial to the host. Centromeric sequences are polymorphic, yet the centromere represents a functionally highly conserved cytological structure [12,60]. Most centromeric sequences are repetitive in nature. While centromeric satellites evolve rapidly at the sequence level (to the extent that they are largely species-specific) [61-63], centromeric retrotransposons appear to evolve more slowly. However, as the centromeres are assumed to be determined more epigenetically than genetically [64], it is unlikely that the centromeric retrotransposon sequence itself can be a direct determinant of centromere identity and function. Instead, it is probable that these repetitive sequences help to produce a conducive genomic environment for the establishment of centromeric chromatin. The promoters of centromeric retrotransposons may be important not only for their own transcription but also for the transcription of adjacent sequences as suggested by Jiang [12]. While it remains to be confirmed that their transcription is required for the deposition of CenH3 into the centromere, it does seem clear that transcripts of centromeric repeats do play some role in determining the integrity of centromeric chromatin and pericentromeric heterochromatin [45,65-67]. CRM element transcripts remain bound to CenH3 chromatin, suggesting that they have a stabilizing role in the structure of the maize centromere [34]. All centromeric retrotransposons tested to date are actively transcribed [26,34,35] (Additional file 5: Transcription of centromeric retrotransposons), so it is reasonable to suggest that their function is similar to that of CRM. The outer centromeric repeats in the pericentromeric heterochromatin of fission yeast (*Saccharomyces pombe*) are required for the RNAi-mediated formation of heterochromatin necessary for the establishment of CENP-A (a synonym for CenH3) chromatin in the core domain [44,68]. A portion of the centromeric retrotransposons is also associated with the heterochromatin mark H3K9me2, and at least some of their transcripts are processed via the RNAi pathway [35]. However, although the dependence on RNAi of both heterochromatin formation and centromere function has been demonstrated repeatedly [69-77], defective cell division has not as yet been associated with RNAi mutants in plants [78]. As the production of small RNA derived from centromeric retrotransposon transcripts was not compromised in RNAi mutants (Additional file 5: Transcription of centromeric retrotransposons), the absence of this predicted phenotype in these mutants may reflect a sufficient level of redundancy in the RNAi machinery. However, considering the very low frequency of small RNA sequences, we cannot exclude the possibility that they are merely an artefact of high-throughput sequencing. Therefore, it remains an open question both whether RNAi plays an important role in the regulation of centromeric retrotransposons and whether it is required for normal centromere function in plants.

## Conclusions

Although centromeric retrotransposons were classified as a CRM clade of chromoviruses, our results show that genuinely centromeric retrotransposons represent only a fraction of this clade, which is referred to as group A in this paper. All tested elements from this group have centromeric localization, and most of them contain the CR motif at the C-terminus of their integrase. This motif is crucial for centromere targeting, and its N-terminal part is relatively well conserved even among evolutionarily distant species. Some chromoviruses containing altered sequences of the CR motif or lacking the CR motif also have centromeric localization. It remains unclear, however, whether their localization in centromeres is a result of centromere targeting or some other mechanisms.

The genuinely centromeric retrotransposons are present in both major angiosperm groups (mono- and dicotyledonous species), but have not been found in the gymnosperms and evolutionarily older species. They represent the only relatively conserved component within highly diverse sequences of plant centromeres. Their transpositional activity contributes to high evolutionary dynamics of centromeres by generating new insertions which may be further subjected to illegitimate and unequal homologous recombination. In addition, their transcriptional activity is consistent with the notion that the transcription of centromeric retrotransposons has a role in normal centromere function.

## Methods

### Plant material

Seeds of pea (*Pisum sativum*) cv. Carrera were obtained from Osiva Boršov (Boršov nad Vltavou, Czech Republic). Seeds of barrel medic (*Medicago truncatula*) cv. Jemalong were obtained from the Crop Research Institute (Prague-Ruzyně, Czech Republic). Seeds of white campion (*Silene latifolia*) were obtained from the Institute of Biophysics (Brno, Czech Republic). Seeds of Norway spruce (*Picea abies*) were harvested from natural stands at Strážkovice, Czech Republic. Banana (*Musa acuminata *cv. Calcutta 4 ITC 0249) plants were received from the International Transit Centre, Katholieke Universiteit (Leuven, Belgium), and grape (*Vitis vinifera*) cv. Pinot Noir plants were obtained from N.O.S. (Nepomuk, Czech Republic). Black cottonwood (*Populus trichocarpa*) cuttings were a gift from the Silva Tarouca Research Institute for Landscape and Ornamental Gardening (Průhonice, Czech Republic).

### *In silico *discovery of centromeric retrotransposons and sequence analysis

The *in silico *search strategy depended on the reliable discrimination of CRM chromoviruses from other LTR retrotransposons on the basis of their RT domain protein sequence [27,79]. Thus, all green plant (Viridiplantae) sequences available in the GenBank database were queried with the RIRE7 RT domain using TBLASTN [80,81], with an *e*-value threshold of 1 *e*^-5^. Rice sequences were excluded because the CRR elements have already been well characterized [17]. Full-size elements were identified among the resulting hits using LTR_Finder [82]. Elements from different species, elements which could not be aligned with the others over the whole length of their sequencesand elements sharing less than 70% similarity in the LTR were classified as distinct families. The relaxed TBLASTN stringency generated a diverse set of full-length retrotransposons containing elements from various Ty3/gypsy lineages, and BLASTX was applied to compare their sequences with a comprehensive database of RT domains extracted from all the major groups of plant Ty3/gypsy retrotransposons (data not shown). Only elements which had the best hits for some of the previously described CRM members were retained. As the best hit-based criteria could theoretically have resulted in the selection of chromovirus elements related to, but not necessarily falling within, the CRM clade, a phylogenetic analysis was carried out to clarify the relationships between the various elements. An additional search was conducted of 454-originated sequence data obtained from pea [42], white campion (J. Macas, E. Kejnovský, P. Novák, P. Neumann, A. Koblížková, B. Vyskot, unpublished data) and banana [53]. Contigs assembled from these sequence reads according to the method described by Macas *et al. *[42] were used to identify RT domains as delineated above. *De novo *full-length or nearly full-length sequences of these elements in white campion and pea were obtained from, respectively, bacterial artificial chromosome (BAC) clones and sequenced amplicons. Banana full-length elements corresponding to 454-generated sequences were already represented in GenBank. Although for the majority of the similarity searches we used TBLASTN, some searches were performed at the protein level using programs implemented in either HMMER [83,84] or MEME [85,86]. Sequence analysis was conducted using software within the EMBOSS or Staden packages [87,88], multiple alignments were performed using Clustal X [89] or Muscle [90] software, and pairwise ones were performed using the Stretcher program [91]. Protein domains were identified by searching the Conserved Domains Database with RPS-BLAST [92], and by searching a local database with BLASTP and BLASTX [81]. Phylogenetic analyses relied on a neighbor-joining method using observed evolutionary distances implemented in the SeaView program [93]. Bootstrap values were calculated from 1,000 replications. Phylogenetic trees were drawn and edited using the iTOL [94] and FigTree [95] programs. The timing of individual insertion events was estimated on the basis of comparisons between 5' and 3' LTRs as described by Liu *et al. *[13]. Sequence logos were generated using the WebLogo tool[96]. The distribution of BLAST hits across the whole genome sequence was visualized using the NCBI MapViewer [97]. Small RNA sequences originating from centromeric retrotransposons were identified using BLASTN searches.

### PCR, cloning, sequencing and hybridization

The sequences of all the PCR primers used for retrotransposon amplification and cloning are listed in Additional file 6: PCR primer sequences and targets. Longer fragments were amplified using LA DNA polymerase (Top-Bio, Prague, Czech Republic). Each 30 μl of PCR contained 1 × PCR buffer, 0.2 mM deoxyribonucleotide triphosphate (dNTP), 0.3 μM concentrations of each primer, 2% (wt/vol) dimethyl sulfoxide, 0.3 U of LA DNA polymerase and 150 ng of template. The reaction profile included 35 cycles of 15 seconds at 94°C, 30 seconds at 60°C, and 7 minutes at 68°C, preceded by initial denaturation step (94°C for 60 seconds) and followed by a final extension step (10 minutes at 68°C). Shorter fragments were amplified using Platinum *Taq *DNA Polymerase (Invitrogen, Carlsbad, CA, USA). Here each 25 μl of PCR contained 1 × PCR buffer, 0.2 mM dNTP, 0.2 μM concentrations of each primer, 1.5 mM MgCl_2_, 1 U of Platinum *Taq *DNA Polymerase and 5 ng of template. The reaction profile included 35 cycles of 30 seconds at 94°C, 30 seconds at 55°C, and 1 to 3 minutes at 72°C, preceded by initial denaturation (3 minutes at 94°C) and followed by a final extension step (10 minutes at 72°C). All PCR products were cloned into the pCR4 TOPO plasmid (Invitrogen). The resulting clones were either fully (cID58-2 and cID58-6) or partially sequenced to verify that they contained the intended insert. The sequences of the two fully sequenced inserts have been deposited in GenBank [GenBank:GU136551 and GenBank:GU136552]. A complete list of the clones used in this work is provided in Additional file 6: PCR primer sequences and targets.

A set of 20,000 white campion BAC clones (A. Widmer, unpublished data) was spotted onto a filter and screened by independent hybridizations with α-[^32^P]-dATP-labeled cID51-1 and cID51-2 (Prime-It II Random Primer Labeling Kit; Stratagene, La Jolla, CA, USA). The hybridization method used was the one described by Yang *et al. *[98], which was followed by a high-stringency wash in 0.1 × saline-sodium citrate (SSC) buffer and 0.1% sodium dodecyl sulfate at 65°C. Clones hybridizing strongly with both probes were isolated, and the presence of centromeric elements was verified by PCR. BAC clone BAC105E4 was sequenced using GS FLX technology (454 Life Sciences/Roche, Branford, CT, USA) to a depth of 20 × at GATC Biotech AG (Konstanz, Germany). Reads (mean length, 250 bp) were assembled into contigs using CAP3 [99]. The sequences of the SilL1 and SilL2 retrotransposons present in this BAC clone have been deposited in GenBank [GenBank:GU136549 and GenBank:GU136550]. Copy numbers were estimated for the Spdl-like sequence (clones cID79-1 and cID81-4), VitV1 (cID73-4 and cID74-1), VitV2 (cID91-2) and VitV3 (cID90-10) as described elsewhere [43]. These estimates were based on the published 1C genome sizes of grape (0.43 pg [100]) and Norway spruce (18.6 pg [101]).

### Fluorescence *in situ *hybridization

Root meristems were obtained from young seedlings (barrel medic, Norway spruce, pea, white campion) or plants (banana, black cottonwood, grape). The accumulation of meristematic cells at metaphase for banana, pea, Norway spruce and white campion was carried out following the methods described by, respectively, Doleželová *et al. *[102], Neumann *et al. *[43], Űberall *et al. *[103] and Kejnovský *et al. *[11], while for the remaining species, mitotic metaphases were accumulated by treatment of the roots with 2.5 μM amiprophos-methyl (in 1 × Hoagland's solution) for 2 hours at room temperature. Mitotic spreads of barrel medic, Norway spruce, pea, black cottonwood and grape chromosomes were made using a conventional squashing method, followed by RNase A and pepsin treatment [104]. FISH probes for these species were labeled by nick translation incorporation of biotin-deoxyuridine triphosphatase (biotin-dUTP) [104] into a plasmid containing a retrotransposon insert. The following clones were used as sources of FISH probes: cID58-2 (PiSat1), cID64-3 plus cID68-2 (MedT1, MedT2), cID79-1 plus cID81-4 (Spdl-like sequences), cID85-11 (PopT2), cID91-2 (VitV2), cID90-10 (VitV3), cID73-4 plus cID74-1 (both VitV1) and Psat32 (partial sequence of the Peabody retrotransposon [43]). FISH hybridization was performed overnight at 28°C, followed by a posthybridization wash first in 2 × SSC at 32°C for 5 minutes and then in 50% (vol/vol) formamide in 2 × SSC at 32°C for 10 minutes. Biotinylated probes were detected as described by Leitch *et al. *[104] using fluorescein-avidin DN and biotinylated anti-avidin D. Chromosomes were counterstained with 4',6-diamidino-2-phenylindole. Images were captured with a DS-Qi1Mc cooled camera (Nikon, Tokyo, Japan) and analyzed using NIS Elements 3.0 software (Laboratory Imaging, Prague, Czech Republic). For white campion, chromosome preparation, probe labeling, hybridization and signal detection followed the methods described by Kejnovský *et al. *[11]. Probes consisting of SilL1 and SilL2 LTR fragments were amplified from BAC105E4 (for primer sequences, see Additional file 6: PCR primer sequences and targets). Chromosome preparations of banana and the subsequent hybridization and signal detection procedures followed the methods described by Doleželová *et al. *[102]. The MusA1 probe was PCR-labeled with biotin-dUTP from a template of clone cID53-1 DNA.

### RT-PCR

Total RNA was isolated from leaves using TRIzol reagent (Invitrogen) and treated with DNase I (Ambion, Austin, TX, USA). First-strand synthesis was achieved using a SuperScript III First-Strand Synthesis System for RT-PCR kit (Invitrogen) according to the manufacturer's recommendations and employing random hexamers as primers. A sample of 5 ng of the resulting cDNA was used as a template for 25 μl of PCR containing 1 × PCR buffer, 0.2 mM dNTP, 0.2 μM concentrations of each primer, 1.5 mM MgCl_2 _and 1 U of Platinum *Taq *DNA Polymerase (Invitrogen). The amplification regime included 35 cycles of 30 sec at 94°C, 50 sec at 55°C, and 1-3 minutes at 72°C, preceded by initial denaturation (3 min at 94°C) and followed by a final extension step (10 min at 72°C). All relevant primer sequences are given in Additional file 6: PCR primer sequences and targets.

## Competing interests

The authors declare that they have no competing interests.

## Authors' contributions

PN and JM designed the study. PN carried out bioinformatics analyses and participated in some experiments. JM, EK, JD and EH were involved in 454 sequencing. JM processed sequence data from 454 sequencing. AN carried out fluorescent *in situ *hybridization (FISH) experiments in pea, black cottonwood, barrel medic, grape and Norway spruce. EK and EH carried out FISH in white campion and banana, respectively. AK participated in cloning and sequencing experiments. AW constructed the bacterial artificial chromosome (BAC) cloning library of white campion. RH and EK screened the BAC library and sequenced the BAC clone BAC105E4. PN and JM drafted the manuscript. All authors read and approved the final manuscript.

## Supplementary Material

Additional file 1 **Origin and structural features of sequences used in this work**. **(A) **Origin and **(B) **sequence and structural features of CRM clade chromoviruses. **(C) **Elements belonging to the Tekay, Reina and Galadriel clades. Click here for file

Additional file 2 **CRM sequences used in this study**. Click here for file

Additional file 3 **Alignment of reverse transcriptase domains**. Click here for file

Additional file 4 **Dot plot comparison of full-length CRM elements**. The elements are ordered according to group and plant family. Each family is represented by one element. Click here for file

Additional file 5 **Transcription of centromeric retrotransposons. (A) **Reverse transcriptase-polymerase chain reaction (RT-PCR) analysis using primer pairs amplifying the RT coding domain (see Additional file 6, PCR primer sequences and targets). The three templates shown are reverse-transcribed RNA (+), nontreated RNA (-) and genomic DNA (g). **(B) **Size distribution of centromeric retrotransposon-derived small RNA. **(C) **The abundance of centromeric retrotransposon-derived small RNA in various tissues and in *Arabidopsis thaliana *RNA interference mutants. Data recovered from two different Gene Expression Omnibus accessions (http://www.ncbi.nlm.nih.gov/geo/) are indicated by black or gray columns. Columns containing data obtained from RNAi mutants are indicated by hatched bars, and the identity of the defective genes is indicated. The small RNA abundance was normalized against the total number of small RNA. TPQ, number of occurrences per quarter million. Click here for file

Additional file 6 **Polymerase chain reaction primer sequences and targets**. Click here for file
