# Supplementary figures and images for: Plant centromeric retrotransposons: a structural and cytogenetic perspective
Source: Mob DNA. 2011 Mar 3;2:4. doi: 10.1186/1759-8753-2-4 (PMC3059260; doi:10.1186/1759-8753-2-4)

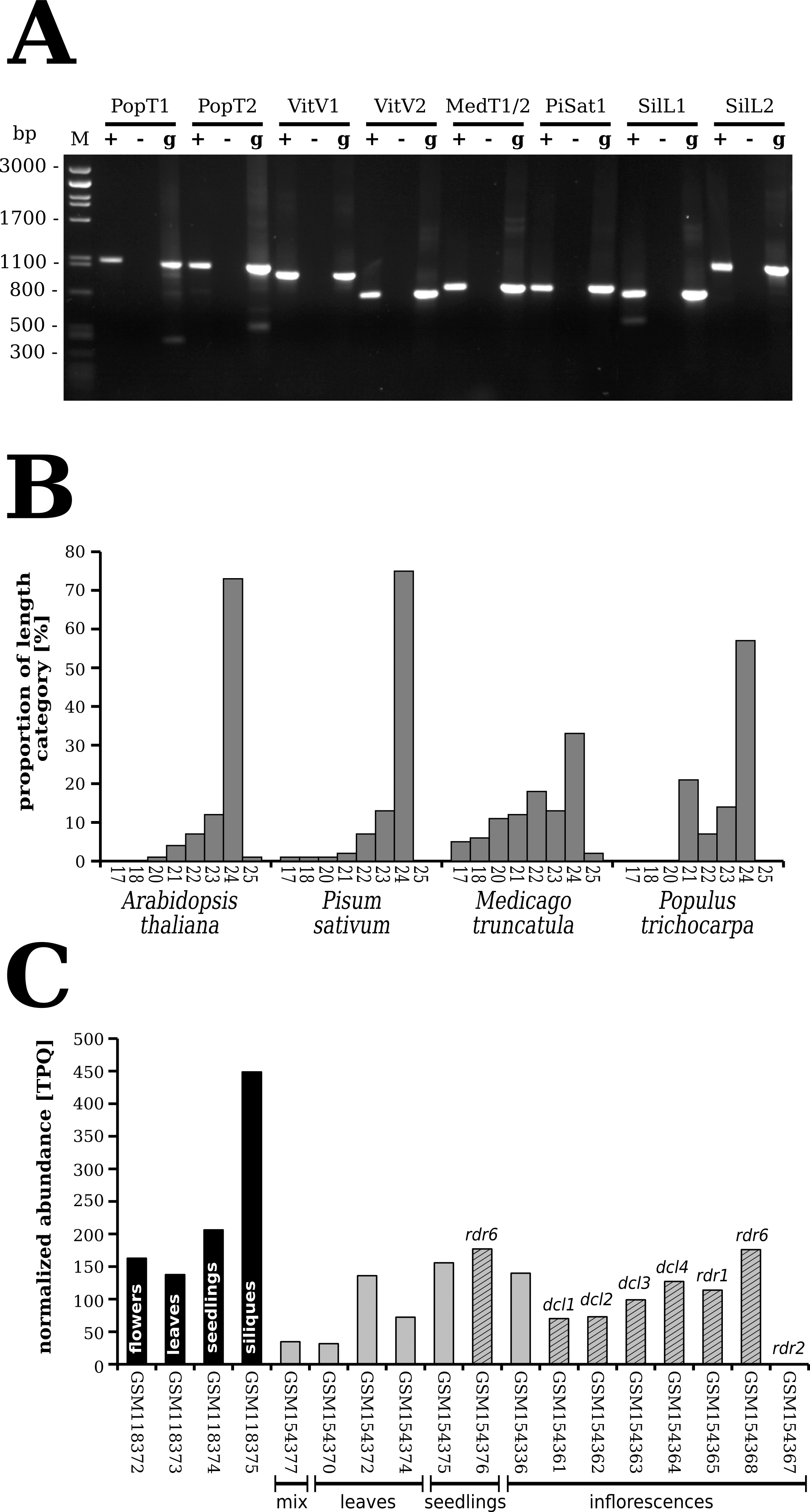

Supplement: Additional file 5 — Transcription of centromeric retrotransposons. (A) Reverse transcriptase-polymerase chain reaction (RT-PCR) analysis using primer pairs amplifying the RT coding domain (see Additional file 6, PCR primer sequences and targets). The three templates shown are reverse-transcribed RNA (+), nontreated RNA (-) and genomic DNA (g). (B) Size distribution of centromeric retrotransposon-derived small RNA. (C) The abundance of centromeric retrotransposon-derived small RNA in various tissues and in Arabidopsis thaliana RNA interference mutants. Data recovered from two different Gene Expression Omnibus accessions (http://www.ncbi.nlm.nih.gov/geo/) are indicated by black or gray columns. Columns containing data obtained from RNAi mutants are indicated by hatched bars, and the identity of the defective genes is indicated. The small RNA abundance was normalized against the total number of small RNA. TPQ, number of occurrences per quarter million. [file 1759-8753-2-4-S5.TIFF]
