# Supplementary material for: Plant centromeric retrotransposons: a structural and cytogenetic perspective
Source: Mob DNA. 2011 Mar 3;2:4. doi: 10.1186/1759-8753-2-4 (PMC3059260; doi:10.1186/1759-8753-2-4)
Supplement: Additional file 4 — Dot plot comparison of full-length CRM elements. The elements are ordered according to group and plant family. Each family is represented by one element. [file 1759-8753-2-4-S4.PDF]

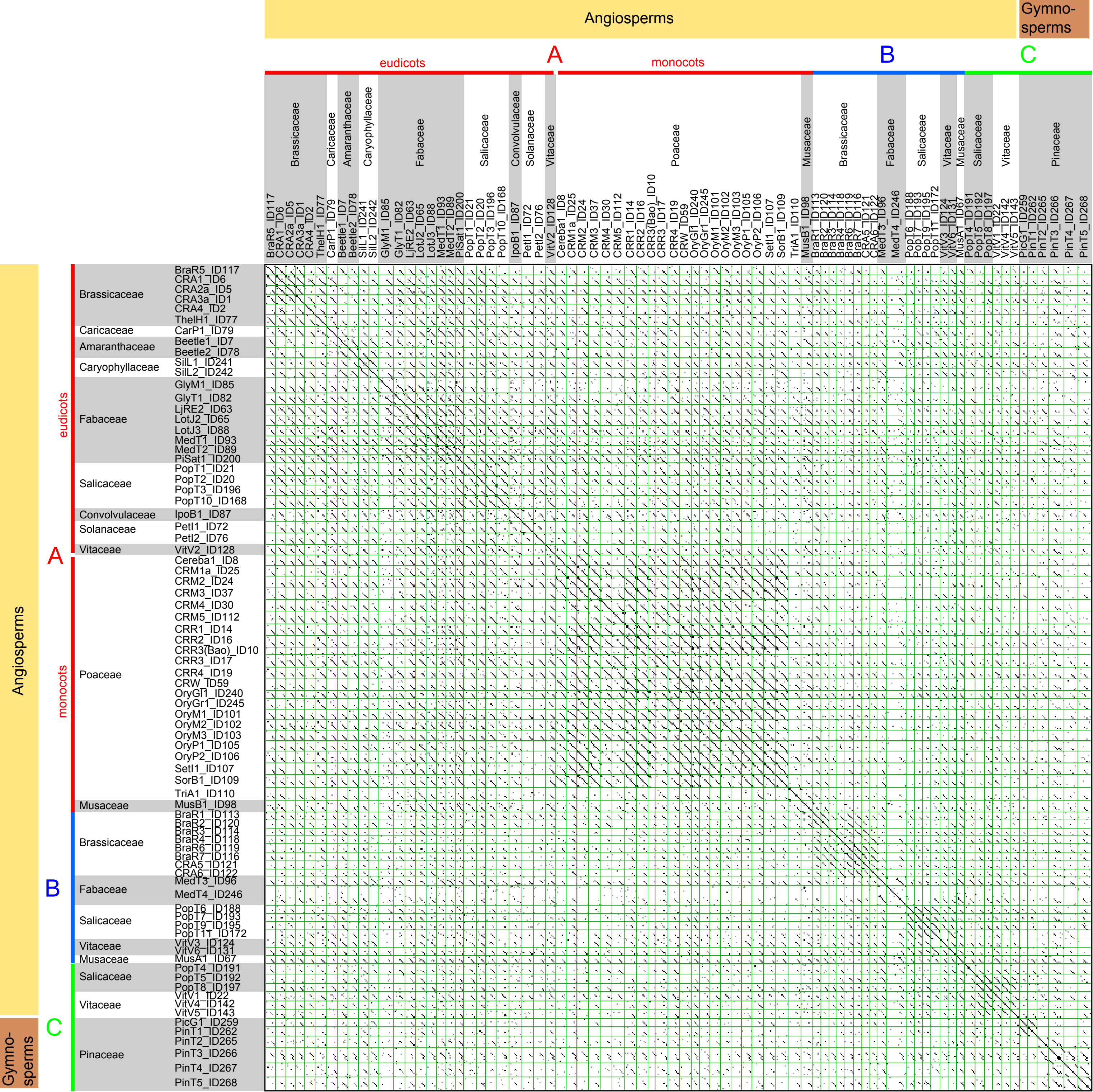
Angiosperms
Gymno-sperms
A
B
C
eudicots
monocots
Brassicaceae
BraR5_ID117
CRA1_ID6
CRA2a_ID5
CRA3a_ID1
CRA4_ID2
ThelH1_ID77
Caricaceae
CarP1_ID79
Amaranthaceae
Beetle1_ID7
Beetle2_ID78
Caryophyllaceae
SilL1_ID241
SilL2_ID242
Fabaceae
GlyM1_ID85
GlyT1_ID82
LjRE2_ID63
LotJ2_ID65
LotJ3_ID88
MedT1_ID93
MedT2_ID89
PiSat1_ID200
Salicaceae
PopT1_ID21
PopT2_ID20
PopT3_ID196
PopT10_ID168
Convolvulaceae
IpoB1_ID87
Solanaceae
PetI1_ID72
PetI2_ID76
Vitaceae
VitV2_ID128
Poaceae
Cereba1_ID8
CRM1a_ID25
CRM2_ID24
CRM3_ID37
CRM4_ID30
CRM5_ID112
CRR1_ID14
CRR2_ID16
CRR3(Bao)_ID10
CRR3_ID17
CRR4_ID19
CRW_ID59
OryGl1_ID240
OryGr1_ID245
OryM1_ID101
OryM2_ID102
OryM3_ID103
OryP1_ID105
OryP2_ID106
SetI1_ID107
SorB1_ID109
TriA1_ID110
Musaceae
MusB1_ID98
Brassicaceae
BraR1_ID113
BraR2_ID120
BraR3_ID114
BraR4_ID118
BraR6_ID119
BraR7_ID116
CRA5_ID121
CRA6_ID122
Fabaceae
MedT3_ID96
MedT4_ID246
Salicaceae
PopT6_ID188
PopT7_ID193
PopT9_ID195
PopT11_ID172
Vitaceae
VitV3_ID124
VitV6_ID131
Musaceae
MusA1_ID67
Salicaceae
PopT4_ID191
PopT5_ID192
PopT8_ID197
Vitaceae
VitV1_ID22
VitV4_ID142
VitV5_ID143
Pinaceae
PicG1_ID259
PinT1_ID262
PinT2_ID265
PinT3_ID266
PinT4_ID267
PinT5_ID268
